# Examining Linguistic Differences in Electronic Health Records for Diverse Patients With Diabetes: Natural Language Processing Analysis

**DOI:** 10.2196/50428

**Published:** 2024-05-23

**Authors:** Isabel Bilotta, Scott Tonidandel, Winston R Liaw, Eden King, Diana N Carvajal, Ayana Taylor, Julie Thamby, Yang Xiang, Cui Tao, Michael Hansen

**Affiliations:** 1 Deutser, Houston, TX, United States; 2 Belk College of Business, University of North Carolina at Charlotte, Charlotte, NC, United States; 3 Department of Health Systems and Population Health Sciences, University of Houston Tilman J. Fertitta Family College of Medicine, Houston, TX, United States; 4 Department of Psychological Sciences, Rice University, Houston, TX, United States; 5 Department of Family & Community Medicine, University of Maryland, Baltimore, MD, United States; 6 Department of Physical Medicine and Rehabilitation, University of California, Los Angeles, Los Angeles, CA, United States; 7 Duke University School of Medicine, Durham, NC, United States; 8 Peng Cheng Laboratory, Shenzhen, China; 9 Department of Artificial Intelligence and Informatics, Mayo Clinic, Jacksonville, FL, United States; 10 Depatment of Family and Community Medicine, Baylor College of Medicine, Houston, TX, United States

**Keywords:** bias, sociodemographic factors, health care disparities, natural language processing, sentiment analysis, diabetes, electronic health record, racial, ethnic, diversity, Hispanic, medical interaction

## Abstract

**Background:**

Individuals from minoritized racial and ethnic backgrounds experience pernicious and pervasive health disparities that have emerged, in part, from clinician bias.

**Objective:**

We used a natural language processing approach to examine whether linguistic markers in electronic health record (EHR) notes differ based on the race and ethnicity of the patient. To validate this methodological approach, we also assessed the extent to which clinicians perceive linguistic markers to be indicative of bias.

**Methods:**

In this cross-sectional study, we extracted EHR notes for patients who were aged 18 years or older; had more than 5 years of diabetes diagnosis codes; and received care between 2006 and 2014 from family physicians, general internists, or endocrinologists practicing in an urban, academic network of clinics. The race and ethnicity of patients were defined as *White non-Hispanic*, *Black non-Hispanic*, or *Hispanic or Latino*. We hypothesized that Sentiment Analysis and Social Cognition Engine (SEANCE) components (ie, negative adjectives, positive adjectives, joy words, fear and disgust words, politics words, respect words, trust verbs, and well-being words) and mean word count would be indicators of bias if racial differences emerged. We performed linear mixed effects analyses to examine the relationship between the outcomes of interest (the SEANCE components and word count) and patient race and ethnicity, controlling for patient age. To validate this approach, we asked clinicians to indicate the extent to which they thought variation in the use of SEANCE language domains for different racial and ethnic groups was reflective of bias in EHR notes.

**Results:**

We examined EHR notes (n=12,905) of Black non-Hispanic, White non-Hispanic, and Hispanic or Latino patients (n=1562), who were seen by 281 physicians. A total of 27 clinicians participated in the validation study. In terms of bias, participants rated negative adjectives as 8.63 (SD 2.06), fear and disgust words as 8.11 (SD 2.15), and positive adjectives as 7.93 (SD 2.46) on a scale of 1 to 10, with 10 being extremely indicative of bias. Notes for Black non-Hispanic patients contained significantly more negative adjectives (coefficient 0.07, SE 0.02) and significantly more fear and disgust words (coefficient 0.007, SE 0.002) than those for White non-Hispanic patients. The notes for Hispanic or Latino patients included significantly fewer positive adjectives (coefficient −0.02, SE 0.007), trust verbs (coefficient −0.009, SE 0.004), and joy words (coefficient −0.03, SE 0.01) than those for White non-Hispanic patients.

**Conclusions:**

This approach may enable physicians and researchers to identify and mitigate bias in medical interactions, with the goal of reducing health disparities stemming from bias.

## Introduction

### Background

Language and communication play a significant, if not primary, role in social relations across different cultures [1]. Language has increasingly been recognized as a relevant form of data that describe relations and behavior [2]. One of the most intimate forms of communication between individuals occurs between clinicians and patients during clinical visits. However, these encounters may be undermined by different forms of bias directed toward patients from certain racial and ethnic minority groups [3]. Generally, *bias* refers to an evaluation, decision, perception, or action in favor of or against a person or group compared to another. Bias can be blatant, wherein it is characterized by deliberate actions (eg, racist comments) that are intentionally and overtly discriminatory [4]. Bias can also be subtle, including “actions that are ambiguous in intent to harm, difficult to detect, low in intensity, and often unintentional but are nevertheless deleterious” to targets [4]. Subtle bias by health care clinicians is linked to negative outcomes for racial and ethnic minority patients, particularly Black non-Hispanic and Hispanic or Latino patients [5].

### Race and Racial Bias in Medical Interactions

Health disparities between racial and ethnic groups have historically been attributed to varying levels of socioeconomic status, as well as genetic and biological factors that were thought to predispose groups to different medical conditions. Research has emerged over the past few decades demonstrating that in fact, there is no biological basis for racial and ethnic differences. Humans share 99.9% of their genome, and the 0.1% variation cannot be explained or elucidated by race [6]. Race describes physical traits considered socially significant, and ethnicity denotes a shared cultural heritage, such as language, practices, and beliefs [7]. As such, race and ethnicity are social constructs, and since the landmark report *Unequal Treatment* in 2002 detailed the impact of racial and ethnic discrimination in patient-clinician interactions, research interest in this area has burgeoned [8]. Relative to White non-Hispanic patients, Black non-Hispanic and Hispanic or Latino patients are less likely to ‘‘engender empathic responses from clinicians, establish rapport with clinicians, receive sufficient information, and be encouraged to participate in medical decision making” [9]. A lack of relationship building [10], reduced positive patient and clinician affect [11], decreased patient trust [12], and fewer patient questions [13] are all more likely outcomes for Black non-Hispanic and Hispanic or Latino patients compared to White non-Hispanic patients during medical interactions. Indeed, the 2018 *National Healthcare Disparities Report* revealed that, compared to White non-Hispanic patients, Black non-Hispanic patients receive inferior care on 40% of quality measures, and Hispanic or Latino patients receive worse care on 35% of quality measures, many of which indicate biased and discriminatory behaviors by clinicians [14]. For example, indicators were worse for Black non-Hispanic and Hispanic or Latino patients than White non-Hispanic patients for measures such as “physicians sometimes or never showed respect for what they had to say” and “physicians sometimes or never spent enough time with them” [14]. Black non-Hispanic and Hispanic or Latino patients are more likely to report racial and ethnic bias and discrimination during medical encounters compared to White non-Hispanic patients [15]. Yet, less is known about the manifestations and details of such experiences during the clinician-patient interaction [16] and whether racial and ethnic discrepancies in care can be observed in the content of electronic health records (EHRs). Similar to the thesis described in *Unequal Treatment*, we hypothesized that the mitigation of bias at the clinician level is needed to improve patient outcomes for diverse racial and ethnic populations and narrow the disparities gap. To address bias, researchers need to understand how to measure its existence, and clinicians need to be informed of its manifestations.

### Research Contributions

Bias can have many forms—blatant, subtle, malevolent, or benevolent—all of which can be indicated by language. With increasing access to EHR documentation and advances in natural language processing, we may be better equipped to identify differences in clinician encounters with patients of diverse racial and ethnic backgrounds. This study searched for linguistic discrepancies in EHRs using a natural language processing approach followed by linear mixed effect model analyses. EHRs are digital summaries of the clinician-patient encounter and include the clinician’s assessment of the interaction, as well as the patient’s health history. Since the clinician is responsible for inputting information, as well as reviewing the information inputted by other care clinicians in the EHR for each patient encounter, the contents of the EHR may be particularly useful in illuminating biases that clinicians hold toward patients of different racial and ethnic backgrounds. Although several studies have indicated that clinician bias occurs, particularly in racially and ethnically discordant interactions (ie, when the patient and clinician are of different racial and ethnic backgrounds), relatively little research has examined the ways in which the clinician may be thinking about the patient and how the clinician’s sentiment and cognitions are reflected in the language of the EHR [8,17]. EHRs can include many years of patient-clinician interactions, with multiple clinicians having access to them, allowing for biases to be passed on and potentially impact future medical decisions.

Our data set contained EHR notes for a large sample of White non-Hispanic, Black non-Hispanic, and Hispanic or Latino patients with diabetes in the Southern United States. The natural language processing tool, Sentiment Analysis and Social Cognition Engine (SEANCE), was applied to assess multiple linguistic markers in the EHR text [18,19]. We then explored whether 8 of the 20 SEANCE components (see Table 1) differed for patients of different races and ethnicities [20,21].

**Table 1.** Description of SEANCE^a^ components.

Component label	Indices, n^b^	Key indices^c^	Language examples
Negative adjectives	18	NRC^d^ negative adjectives, NRC disgust adjectives, NRC anger adjectives, GI^e^ negative adjectives, and Hu-Liu^f^ negative adjectives	Unkind, bad, cruel, hurtful, and intolerant
Positive adjectives	9	Hu-Liu positive adjectives, VADER^g^ positive adjectives, GI positive adjectives, and Lasswell^h^ positive affect adjectives	Supportive, kind, great, and nice
Joy words	8	NRC joy adjectives, NRC anticipation adjectives, and NRC surprise adjectives	Admiration, advocacy, elated, glad, liking, and pleased
Fear and disgust words	8	NRC disgust nouns, NRC negative nouns, NRC fear nouns, and NRC anger nouns	Abnormal, adverse, attack, cringe, criticize, distress, intimidate, unequal, and stigma
Politics words	7	GI politics nouns and Lasswell power nouns	Alliance, ally, authorize, civil, concession, consent, and oppose
Respect words	4	Lasswell respect nouns	Status, honor, recognition, and prestige
Trust verbs	5	NRC trust verbs, NRC joy verbs, and NRC positive verbs	Affirm, advise, confide, and cooperating
Well-being words	4	Lasswell well-being physical nouns and Lasswell well-being total nouns	Alive, ambulance, adjust, afraid, blood, clinic, and nutrition

a SEANCE: Sentiment Analysis and Social Cognition Engine.

b Indices refer to the number of dictionary lists from which the component was developed.

c The key indices came from the following dictionary lists: NRC Emotion Lexicon [18,22], the Harvard-IV dictionary list used by the General Inquirer [23], the Hu-Liu polarity word lists [22,23], the Valence Aware Dictionary and Sentiment Reasoner [24], the Lasswell dictionary lists [25,26], and the Geneva Affect Label Coder database [27]. For a thorough review of the SEANCE indices and corresponding dictionaries, see Crossley et al [18].

d NRC: NRC Emotion Lexicon.

e GI: General Inquirer.

f Hu-Liu: Hu-Liu polarity word lists.

g VADER: Valence Aware Dictionary and Sentiment Reasoner.

h Lasswell: Lasswell dictionary lists.

We hypothesized that the SEANCE components for negative adjectives, positive adjectives, joy words, fear and disgust words, politics words, respect words, trust verbs, and well-being words and the mean word count in the notes would be indicators of bias, as these concepts have been linked to bias in nonmedical contexts. Ng’s [28] review of linguistic racial bias in verbiage offers the rationale for our choice of fear and disgust words, politics words, respect words, and trust verbs as indicators of bias, whereas the work of Li et al [29] examining gender differences in standardized writing assessment provides further support for our use of SEANCE as a tool for examining biases in language. We selected positive and negative adjectives, well-being words, politics words, and word count indicators as prior research demonstrates that clinicians may be less likely to establish rapport and provide appropriate medications and are more inclined to show negative attitudes and be dismissive toward Black non-Hispanic and Hispanic or Latino patients as a result of their unconscious racial and ethnic biases [30-33].

Specifically, we investigated which aspects of communication differ and whether differences are indicative of biased interactions. Any systematic variation in language can convey differential perceptions, attitudes, and expectations. For example, words such as “resistant” or “non-compliant” could reflect bias if (all else being equal) they tend to be used more to reflect people from some racial or ethnic backgrounds than others. This work aimed to elucidate for clinicians and researchers where discrepancies in communication emerge in the EHR and whether these differences are indicative of racial and ethnic bias. We also assessed the extent to which clinicians perceive linguistic markers to be indicative of bias.

## Methods

### Sample

This was a cross-sectional study using EHR-derived physician notation of outpatient clinical encounters. We extracted EHR encounters (n=15,460) for patients (n=1647) who were aged 18 years or older; had more than 5 years of diabetes diagnosis codes; and received care between 2006 and 2014 from family physicians, general internists, or endocrinologists practicing in an urban, academic network of clinics. We chose this disease because of its high prevalence (11.3% in the United States) and chose to examine outpatient visits because of the relative scope of annual outpatient visits (1 billion) relative to hospital admissions (32 million) [34-36]. The demographic variables collected were patient race and ethnicity, sex, and age. The race and ethnicity of patients were defined as *White non-Hispanic*, *Black non-Hispanic*, or *Hispanic or Latino* (see Table 2 for a summary of patient demographics).

**Table 2.** Patient demographics of the final sample.

Variable		Value (n=1562)
**Age (years)**		
	Mean (SD)	68.74 (13.76)
	Range	20-102
	Median (IQR)	69 (61-78)
**Sex, n (%)**		
	Female	871 (55.74)
	Male	691 (44.26)
**Race and ethnicity, n (%)**		
	White non-Hispanic	682 (43.66)
	Black non-Hispanic	755 (48.34)
	Hispanic or Latino	125 (8)

### SEANCE Algorithm

SEANCE is a lexical scoring algorithm that includes over 200 word vectors (also referred to as indices or features) designed to assess sentiment, cognition, and social order, which were developed from preexisting and widely used databases such as EmoLex and SenticNet [22,37]. In addition to the core indices, SEANCE allows for several customized indices, including filtering for particular parts of speech and controlling for instances of negation [18]. Since SEANCE computes such a large quantity of indices, Crossley et al [18] developed 20 components from all the indices using principal component analysis (PCA) [18]. These components are essentially clusters of related indices in SEANCE and allow users to interpret the SEANCE output at a more macro level. This process enabled them to summarize the SEANCE indices into a smaller and more interpretable set of variables. In the PCA by Crossley et al [18], they retained even the smallest components, setting a conservative cutoff point for inclusion (ie, 1% for variance explained by each component). The analyses for this research were run on a subset of 8 of the 20 *components* that Crossley et al [18] developed. We selected these 8 components a priori (see Table 1 for a description of the selected components).

We chose SEANCE instead of other natural language processing tools, such as Linguistic Inquiry and Word Count (LIWC), because it contains a larger number of core indices taken from multiple lexicons, as well as 20 components, and is based on the most recent improvements in sentiment analysis [18]. In their validation of SEANCE, Crossley et al [18] found that SEANCE components demonstrated significantly greater accuracy than LIWC indices (*P*<.001) for 3 of the 4 review types examined. In addition to the core indices, SEANCE allows for several customized indices, including filtering for parts of speech (also known as “parts-of-speech tagging”) and controlling for instances of negation, which LIWC does not offer. We analyzed all words in the EHR (ie, *not* single parts of speech), but we controlled for negation. For example, this means that “not good” would be recognized as *not being positive* by SEANCE, as opposed to LIWC, which would see the word “good” and count it as positive.

### Validation of the Sentiment Analysis Approach

To provide validation of the sentiment analysis approach used in this study, we surveyed subject-matter experts in EHR note writing (ie, physicians, physician assistants, and nurse practitioners) to garner their perspectives on the appropriateness of the linguistic components identified in our pilot study as indicators of subtle racial and ethnic bias in EHR notes. The team of researchers for this study included industrial-organizational psychologists who have expertise in bias and discrimination; however, it was also valuable to garner opinions from clinicians who are experts in EHR note writing and who understand the differences in the types of language used. To recruit participants, we used a combination of opportunistic and snowball sampling, starting with individuals within our personal networks. Through a web-based program, we asked participants to indicate the extent to which they thought the language domains (eg, negative adjectives, fear and disgust words, etc) were reflective of bias in EHR notes. Participants were told the following:

*One type of language that could represent bias reflects the amount of NEGATIVE ADJECTIVES contained in the electronic health record. Examples of negative adjectives include “unkind,” “bad,” “harmful,” “intolerant,” and “stupid.” If these kinds of words were used to describe Black or LatinX patients more than White patients, to what extent do you think this would be indicative of racial bias? Please indicate the extent of your agreement on the 1 to 10 scale below.*

The same formatting was used for each of the linguistic components, with component-specific language examples offered so participants understood the types of sentiment that each component was designed to assess.

### Cross-Classified Linear Mixed Effects Models

We used the *lme4* package in R (R Foundation for Statistical Computing) to perform linear mixed effects analyses of the relationships between the outcomes of interest (SEANCE components and word count) and patient race and ethnicity, controlling for patient age. We ran an identical analysis, treating 8 different SEANCE components and the mean word count in the EHR as the dependent variables, while leaving all other variables consistent across the models. The same steps of entering fixed and random effects were applied across all cross-classified linear mixed effects models with different dependent variables (ie, negative adjectives, positive adjectives, well-being words, trust verbs, fear and disgust words, joy words, politics words, respect words, and mean word count).

We first ran a null model with only the random intercepts. We then added random effects and applied a crossed design (vs a traditional nested structure), leading us to have intercepts for physicians and patients. Then, we ran a model with the random intercepts as well as the fixed effects. As fixed effects, we entered *race and ethnicity* and *age* (without an interaction term) into the model. For all models examined, the intercept variation can be attributed primarily to different physicians rather than patients. We used a 95% CI to determine statistical significance. To be more conservative, given that we ran multiple tests, we also computed an additional set of CIs at the 99th percentile.

### Ethical Considerations

We obtained ethics approval from the University of Texas Health Science Center’s Committee for the Protection of Human Subjects (HSC-MS-18-0431) and the Rice University Institutional Review Board (IRB-FY2021-325). Participants consented and received a US $25 gift card after completing the survey. EHR data were deidentified prior to the analysis.

## Results

### Description and Justification for Cross-Classified Analyses

An initial inspection of the data revealed that 2 physicians were extreme outliers, accounting for 16.53% (2555/15,460) of the notes in our sample. To ensure that the overrepresentation of these physicians would not bias the results, we removed those notes from the data set (taking us from our initial sample of 15,460 visits with 283 physicians and 1647 patients to 12,905 visits with 281 physicians and 1562 patients; Table 2). The distribution of visits by patients indicates an average of 8.27 visits per patient with a minimum of 1, a median of 5, and a maximum of 97. Physicians see 11.72 patients on average, with a median of 2 and a maximum of 143, suggesting a skewed distribution. Despite the relatively large number of patients seen by some physicians, these physicians accounted for substantially fewer patient notes than the 2 physicians that were previously removed. Patients see 2.11 physicians on average, with a minimum of 1 and a maximum of 12; however, the distribution suggests that 6.6% (109/1647) of patients saw 5 or more physicians. Moreover, 742 (45.1%) of the 1647 patients saw 1 physician, whereas 119 (7.2%) saw 4 physicians. In our data set, patients can have multiple visits to a variety of physicians, indicating that patient visits are not nested within physicians. Further, physicians may see different patients with no consistent overlap of patients between physicians, indicating that physicians are not nested within patients. Thus, there is no clear hierarchical nesting of patients within physicians (or vice versa), which suggests that a cross-classified design is more appropriate than a traditional, hierarchical, multilevel model structure.

### Cross-Classified Linear Mixed Effects Model Results

In the negative adjective component model (Table 3), the random effects of patient (σ^2^=0.02) and physician (σ^2^=0.12) indicated that intercept variation in use of negative adjectives is mainly a function of the physician rather than the patient. The physician random effect was over 5 times as large as the random effect for the patient; the intraclass correlation (ICC) for physicians was 0.41 and the ICC for patients was 0.07 (ICC_total_=0.481). This pattern of results in random effects and ICC values for patients and physicians was consistent across the other 8 models. Overall, 2 of the 5 relationships (ie, the significant difference in positive adjectives for Hispanic or Latino and White non-Hispanic patient notes, and the significant difference in trust verbs for Hispanic or Latino and White non-Hispanic patient notes) that were previously significant at the 95th percentile had CIs that included zero at the 99th percentile. For 3 of the SEANCE components—well-being, politics, and respect words—and for the overall word count, there was not a statistically significant difference between the 3 races and ethnicities. In contrast, for all the other remaining SEANCE components, there was a statistically significant race and ethnicity effect for either Black non-Hispanic or Hispanic or Latino patients relative to White non-Hispanic patients. Specifically, notes for Black non-Hispanic patients contained significantly more negative adjectives and fear and disgust words than those for White non-Hispanic patients. Notes for Hispanic or Latino patients included significantly fewer positive adjectives, trust verbs, and joy words than those for White non-Hispanic patients. As such, across most of the SEANCE components, we observed favoritism of White non-Hispanic patients in terms of note content.

**Table 3.** Fixed effects model results for negative adjectives, positive adjectives, well-being words, trust verbs, joy words, politics words, respect words, fear and disgust words, and word count.

Variables^a^				Negative adjectives	Positive adjectives	Well-being words	Trust verbs	Joy words	Politics words	Respect words	Fear and disgust words	Word count
**Fixed effect estimates**												
	**Age (years)**											
		β (SE)		−0.00 (0.00)	0.00 (0.00)	.0002 (0.00009)	−0.00007 (0.00008)	0.000002 (0.0002)	−0.00009 (0.00004)	−0.00004 (0.00005)	0.000005 (0.00)	−0.43 (0.68)
		95% CI		−0.002 to 0.0003	−0.002 to 0.00	0.0006 to 0.0004^b^	−0.002 to 0.0008	−0.0004 to 0.0004	−0.0002 to −0.000007^b^	−0.0004 to 0.0004	−0.0001 to 0.0002	−1.76 to 0.90
	**Race and ethnicity**											
		**White non-Hispanic (reference)**										
			β (SE)	0.42 (0.05)	−0.24 (0.017)	0.18 (0.007)	0.16 (0.007)	0.32 (0.02)	0.07 (0.003)	0.05 (0.004)	0.17 (0.007)	868.50 (54.45)
			95% CI	0.32 to 0.53	−0.26 to −0.21	0.17 to 0.20	0.14 to 0.17	0.28 to 0.35	0.06 to 0.07	0.04 to 0.05	0.16 to 0.19	761.84 to 975.17
		**Black non-Hispanic**										
			β (SE)	0.07 (0.02)	0.02 (0.004)	0.004 (0.002)	−0.003 (0.002)	−0.01 (0.006)	0.001 (0.001)	−0.001 (0.001)	0.007 (0.002)	20.61 (19.01)
			95% CI	0.04 to 0.11^b^	−0.006 to 0.01	−0.0007 to 0.009	−0.007 to 0.001	−0.02 to 0.0004	−0.001 to 0.004	−0.004 to 0.002	0.003 to 0.01^b^	−16.71 to 57.84
		**Hispanic or Latino**										
			β (SE)	0.02 (0.03)	−0.02 (0.007)	0.002 (0.004)	−0.009 (0.004)	−0.03 (0.01)	−0.0009 (0.003)	0.0006 (0.002)	−0.002 (0.004)	15.73 (32.30)
			95% CI	−0.03 to 0.08	−0.03 to −0.004^b^	−0.007 to 0.01	−0.02 to −0.001^b^	−0.05 to −0.01^b^	−0.005 to 0.003	−0.004 to 0.005	−0.01 to 0.006	−47.61 to 78.98
**Random effects, estimate (SE)**												
	U0 patient			0.02 (0.14)	0.0008 (0.03)	0.0004 (0.02)	0.0002 (0.02)	0.0006 (0.02)	0.00001 (0.004)	0.00002 (0.005)	0.0004 (0.02)	27,878 (167.0)
	U0 physician			0.12 (0.34)	0.006 (0.08)	0.003 (0.05)	0.003 (0.05)	0.02 (0.15)	0.0002 (0.016)	0.0005 (0.02)	0.003 (0.05)	119,489 (345.7)

a Random effects are presented as estimate and SE. For the fixed effect estimates, cell entries are parameter (β) estimates, SE, and 95% CIs. White non-Hispanic was the reference group for race and ethnicity.

b Significant effects based on the 95% CIs.

### Sentiment Analysis Validation

In all, 27 participants completed the surveys (see Multimedia Appendix 1 for the demographics of the participants). On a scale of 1 to 10, with 10 being extremely indicative of bias, participants rated negative adjectives as 8.63 (SD 2.06), fear and disgust words as 8.11 (SD 2.15), positive adjectives as 7.93 (SD 2.46), trust verbs as 7.56 (SD 2.64), and joy words as 6.81 (SD 2.47). The means and SDs for each of the components are reported in Table 4. The results of this preliminary analysis provide support for the validity of the linguistic components as indicators of bias in EHRs, as our sample of clinicians regard them as highly suggestive of bias if used differently for patients of diverse racial and ethnic backgrounds.

**Table 4.** Subject-matter expert assessment of bias based on specific linguistic markers.

Component	Score, mean (SD)^a^	
Negative adjectives	8.63 (2.06)	
Fear and disgust words	8.11 (2.15)	
Positive adjectives	7.93 (2.46)	
Joy words	6.81 (2.47)	
Trust verbs	7.56 (2.64)	
Politics words	7.07 (2.32)	
Respect nouns	7.56 (2.55)	
Well-being words	5.56 (2.55)	
Mean word count	6.11 (2.19)	

a Scale ranges from 1 (*Not at all indicative of bias*) to 10 (*Extremely indicative of bias*).

## Discussion

### Principal Findings

We found that the words that physicians use in EHR notes differ based on the racial and ethnic backgrounds of patients. Specifically, for Black non-Hispanic patients, notes consisted of words that convey negativity, fear, and disgust. When seeing Hispanic or Latino patients, physicians used fewer positive words and were less likely to use words that communicate trust and joy. Our findings are consistent with others who have documented that physicians communicate in the EHR differently (ie, more negatively) when caring for patients from some minority groups [9,17], which may ultimately result in adverse and inequitable health outcomes for patients. Our results also align with other papers that found that stigmatizing language is more commonly used in EHRs for minority populations [38-42]. Those papers used language guidelines [38] and experts [39] to identify stigmatizing language. We came to a similar conclusion by using established language dictionaries and contend that our approach allows for a more comprehensive assessment of language. For example, a prior paper used 15 descriptors [42]. In contrast, our approach encompasses tens of thousands of words, including multiple word lists, positive and negative sentiments, and emotions. Thus, this method does not merely capture the presence or absence of stigmatizing language, but rather offers a broader glimpse of the clinician-patient relationship. Furthermore, the validation survey confirmed that subject-matter experts perceive the types of words included in this study to be indicative of bias when used differentially for patients of diverse racial and ethnic backgrounds. Taken together, these findings indicate that the language used differs for patients based on racial and ethnic backgrounds and that those differences are suggestive of bias. As a result, our paper is the first to use this particular method to examine outpatient, diabetes notes. Since diabetes quality measures already exist, our analysis allows researchers to link bias to differences in quality in future studies [43].

EHR notes are important, although imperfect, assessments of physician attitudes toward their patients. With more and more time now being devoted to EHR documentation, physicians are increasingly burned out, which has led to the adoption of more efficient data entry strategies such as using templates, copy-pasting previous text, and inserting preset language [44,45]. Consequently, notes can be standardized, limiting our ability to assess physician attitudes and subconscious biases toward patients. Despite these caveats, notes remain the definitive and often sole account of what happened in the examination room, and based on these data, Black non-Hispanic and Hispanic or Latino patients are written about differently than White non-Hispanic patients.

The method described in this paper offers a scalable blueprint that provides clinicians with data about their interactions with patients and overcomes limitations of other traditional measures of bias. Existing measures require primary data collection through surveys, videotaped encounters, and confederate observations. Surveys assess perceptions of interactions and are prone to retrospective bias and socially desirable responding, whereas the time-consuming nature of encounters and observations lack scalability and limit the number of clinicians that can receive feedback at any given time. The relevance of alternative measures has also been questioned. For example, critics of the implicit association test have asked whether performance on the test is applicable to real-world contexts [46], which may explain why some change their behavior when confronted with their own biases, whereas others do not [5,47]. In contrast, our method uses data that are automatically and universally collected through the course of delivering care and generated by physicians in actual encounters.

### Limitations

When interpreting our results, several limitations should be considered. First, due to limitations in our data, we are unable to determine which additional team members, including scribes, medical assistants, and residents, contributed to the notes. However, attending physicians are ultimately responsible for the content and have the authority and responsibility to modify language that is inconsistent with their values. Second, we lack information about physicians in this sample and do not have access to physician demographic characteristics (eg, their racial and ethnic backgrounds), although this would be an important next step. We attempted to account for this limitation by comparing language within rather than across physicians. Third, we included all language within notes, including physical exams, medications, and past medical histories. These sections can be guided by templates or not actively entered by physicians. We retained these parts in case the language within these sections contributed to variation. An alternative approach could assess only the history of present illness, assessment, and plan sections of the note and could yield different results. Additional work is needed to determine whether differential word choices reflect attitudes and behaviors toward patients. EHR notes serve a wide range of purposes. They convey medical information to others, remind physicians of their impressions, communicate plans to patients, provide justification for billing codes, and serve as legal evidence [44]. Thus, specific phrases (eg, worsening, uncontrolled, or adherence) may be required for billing, compliance, and legal purposes and may not reflect bias toward patients. Finally, these results may not be generalizable to other conditions. Our findings may be unique to the language used for diabetes care and by clinicians who manage diabetes. Determining whether these results persist for different diseases (eg, cancer, heart disease, and acute injuries) is an important next step.

### Directions for Future Research

Additional research is needed to interpret and provide context for this exploratory work. To determine whether these measures are associated with bias, subject-matter experts could label notes using known patterns of bias (eg, the ratio of collective to personal pronouns, the amount and level of abstraction of speech, and passive vs active voice) [48]. Further research is needed to understand whether biased language in notes reflects biased behaviors during encounters as well as inequitable health outcomes for some racial and ethnic minority populations. Conducting further experiments (eg, with research actors as patients in a mock medical visit) could help determine whether biased language in notes reflects manifestations of bias during encounters (eg, less eye contact, hostile language, or less time spent on education and counseling). If bias is confirmed, we need to determine whether clinicians who use differential language provide worse care and quality for minority patients. Ultimately, this tool may be used to identify and mitigate bias. Future studies should assess whether receiving feedback using this method leads to behavior change and whether changing the language used in EHR notes leads to changes in patient interactions. Although many strategies for reducing bias exist—such as affirming egalitarian goals, seeking common-group identities, perspective taking, and individuation—it is unclear which approach best complements our proposed method [5].

### Conclusion

In this novel, exploratory work, we used natural language processing and found that compared to encounters with White non-Hispanic patients, physicians use language conveying more negativity, fear, and disgust in their encounters with some racial and ethnic minority patients. If confirmed in future studies, these features could be used to make clinicians aware of their biases with the goal of reducing racial and ethnic discrimination and the resulting health inequities.

## Supplementary material

10.2196/50428 Multimedia Appendix 1 Demographics of the validation study participants.

## Abbreviations

EHR: electronic health record
ICC: intraclass correlation
LIWC: Linguistic Inquiry and Word Count
PCA: principal component analysis
SEANCE: Sentiment Analysis and Social Cognition Engine
