# Supplementary material for: Examining Linguistic Differences in Electronic Health Records for Diverse Patients With Diabetes: Natural Language Processing Analysis
Source: JMIR Med Inform. 2024 May 23;12:e50428. doi: 10.2196/50428 (PMC11137426; doi:10.2196/50428)
Supplement: Multimedia Appendix 1 [file medinform-v12-e50428-s001.docx]

**Multimedia Appendix 1.**

Demographics of the Validation Study Participants

| **Race / Ethnicity** | **n** | **Percent** |
| --- | --- | --- |
| White or Caucasian | 12 | 44.4 |
| Asian | 8 | 29.6 |
| Black or African American | 3 | 11.1 |
| Hispanic or Latino | 3 | 11.1 |
| Multiple Races Selected | 1 | 3.7 |
|  |  |  |
|  |  |  |
| **Gender** | **n** | **Percent** |
| Female | 16 | 59.3 |
| Male | 11 | 40.7 |
|  |  |  |
| **Professional Role** | **n** | **Percent** |
| Physician | 25 | 92.6 |
| Nurse Practitioner | 1 | 3.7 |
| Other | 1 | 3.7 |
|  |  |  |
| **Specialty** | **n** | **Percent** |
| Family Medicine | 17 | 63 |
| Pediatrics | 3 | 11.1 |
| Other | 7 | 25.9 |
|  |  |  |
| **Age** | **Mean** | **SD** |
|  | 43.6 | 11.7 |
